# Performance evaluation of the DAAN HCV assay for quantification of hepatitis C virus RNA and its comparison with COBAS AmpliPrep/COBAS TaqMan HCV Quantitative Test, v2.0

**DOI:** 10.1002/jcla.23280

**Published:** 2020-03-13

**Authors:** Yuting He, Yichong Wang, Xuefang Chen, Hao Huang, Jiankai Deng, Peisong Chen, Huang Bin

**Affiliations:** ^1^ Department of Laboratory Medicine The First Affiliated Hospital Sun Yat‐sen University Guangzhou China; ^2^ Nanfang college of Sun Yat‐sen University Guangzhou China

**Keywords:** DAAN HCV assay, HCV RNA quantitative assay, hepatitis C virus RNA, methodology comparison, performance evaluation, Roche Cobas test

## Abstract

**Background:**

The Daan HCV RNA quantitative assay was a recently developed kit with high sensitivity for the detection of HCV RNA. We aimed to evaluate the analytical performance of the Daan HCV RNA quantitative assay and compare it with the COBAS AmpliPrep/COBAS TaqMan HCV Quantitative Test, v2.0.

**Method:**

WHO HCV RNA standard, NIBSC 06/102 standard, and CLSI EP documents were used to evaluate the precision, accuracy, linearity, anti‐interference ability, and cross‐reactivity of the Daan HCV RNA quantitative assay. Overall 198 clinical serum specimens were used to make comparison between the Daan HCV RNA quantitative assay and the Roche Cobas test.

**Results:**

The within‐run precision (S_within_), and total precision (S_total_) for 6.11 log IU/mL, 4.22 log IU/mL, and 2.32 log IU/mL HCV RNA were 0.13 and 0.15, 0.07 and 0.09, and 0.11 and 0.10, respectively. The linear range was 20‐10^8^ IU/mL, and the limit of detection was 15 IU/mL. It did not display any interference with commonly encountered conditions and cross‐reactivity with some common virus. A good agreement was observed between the Daan HCV RNA quantitative assay and the Roche Cobas test.

**Conclusion:**

The Daan HCV RNA quantitative assay has shown satisfactory performances and excellent agreement with COBAS HCV Quantitative Test on clinical specimens with lower cost, which provides an alternative choice for the diagnosis and monitoring of HCV infection in developing countries.

## 1 INTRODUCTION

Hepatitis C virus (HCV) infection remains a significant public health in developing countries, leading to liver cirrhosis, end‐stage liver disease, and hepatocellular carcinoma.^1^ According to the HCV prevalence statistics by the World Health Organization, there were 69.6 million HCV‐infected individuals in 2016 globally.^2^ HCV infection exhibits clinically as acute and chronic hepatitis and is characterized by diffuse liver damage.^3^ Generally, the infection progresses to a chronic state in 80% of patients, many of whom remain asymptomatic for a long time.^4^ It is recommended to measure HCV RNA levels repeatedly during antiviral therapy to determine treatment efficacy and adherence.^5^

Currently, HCV RNA levels can be quantitated by direct measurement of its level in plasma or serum using real‐time polymerase chain reaction (PCR) combined with reverse transcription (RT) or signal amplification methods such as branch DNA(b‐DNA) and reverse transcription loop‐mediated isothermal amplification (RT‐LAMP) assays.^5^, ^6^, ^7^ At present, HCV RNA quantitation has many proprietary systems commercialized by elite companies, such as Roche, Siemens, and Abbott. Meanwhile, HCV PCR quantitative assays are also commercialized by diverse companies such as Hologic, Qiagen, Cepheid, Sacace, Fast‐tracks Diagnostics, and Biocentric. The diagnostic kit for quantification of hepatitis C virus RNA (Daan) is a recently developed and certified kit by China Food and Drug Administration (CFDA). The assay applies the real‐time PCR technology with PCR‐fluorescence probing method. The COBAS AmpliPrep/COBAS TaqMan HCV Quantitative Test, v2.0 (Roche) is a widely used, FDA‐approved HCV assay with excellent performance which is comparable or superior to that of other assays.^8^, ^9^ In this present study, we evaluated the analytical performance of the recently developed and certified diagnostic kit for quantification of hepatitis C virus RNA (Daan), including precision, accuracy, linearity, limit of detection (LOD), interference, and cross‐reactivity study. Then, the HCV RNA quantitative results of the clinical specimens gained using this assay were compared with those gained with the Roche Cobas test.

## 2 MATERIALS AND METHODS

### 2.1 Test devices and instruments

The diagnostic kit for quantification of HCV RNA (Daan) utilizes the real‐time PCR technology with PCR‐fluorescence probing targeting the highly conserved region of HCV and go through a one‐step RT‐PCR to detect the HCV RNA in the plasma or serum. Briefly, HCV RNA was extracted with internal control from 200 μL of serum or plasma and detected by PCR‐fluorescence probing. The internal control is spiked synthetic sequences. It is included in this kit to ensure the sample is extracted properly and there is no carryover of PCR‐inhibitors, reducing the occurrence of false‐negative results. The reaction conditions were in line with the manufacturer's instruction (50°C for 15 minutes, 1 cycle; 95°C for 15 minutes, 1 cycle; 94°C for 15 seconds, 55°C for 45 seconds, 45 cycles; 40°C for 2 seconds, 1 cycle). The extraction of the HCV RNA was performed using automatic nucleic acids extraction apparatus Smart 32 (Daan), and the detection was carried out using the Applied Biosystems 7500 Real‐time fluorescent quantitative PCR instrument (Thermo).

### 2.2 Precision

To evaluate the precision, the World Health Organization (WHO) HCV RNA standard was used which contained 6.11 log IU/mL (1.3 × 10^6^ IU/mL), 4.22 log IU/mL (1.66 × 10^4^ IU/mL), and 2.32 log IU/mL (2.10 × 10^6^ IU/mL), respectively. The American Clinical and Laboratory Standards Institute (CLSI) documents were used as guideline for the design of the experiments. According to CLSI document EP15‐A2,^10^ the three HCV RNA concentrations were tested in five replicates a day, respectively, and the detection lasted for 5 days. Within‐run precision (S_within_), variance term (B), and total precision (S_total_) were calculated and compared with the within‐run precision claimed by the manufacturer (σ_within_) and total precision claimed by the manufacturer (σ_total_). The computational formula for those parameters is shown in Supplementary Material.

### 2.3 Accuracy

To evaluate the accuracy, the 6 log IU/mL HCV RNA reference (National Institute for Biological Standards and Control (NIBSC) 06/102 standard) was used and diluted with HCV‐negative serum to generate five dilutions with nominal HCV RNA concentrations of 10^5^, 10^4^, 10^3^, 10^2^, 50 IU/mL and each dilution was tested in 3 replicates^10^.

### 2.4 Linearity

The high constant concentration sample (2.0 × 10^8^ IU/mL) was diluted with HCV‐negative serum to generate nine dilutions with nominal HCV RNA concentrations of 10^8^, 10^7^, 10^6^, 10^5^, 10^4^, 10^3^, 10^2^, 50, 20 IU/mL, and each dilution was tested in 3 replicates.^11^

### 2.5 Limit of detection (LOD)

3 log IU/mL HCV RNA reference (NIBSC 06/102 standard) was diluted with HCV‐negative serum to generate four dilutions with nominal HCV RNA concentrations of 50, 20, 15, 10 IU/mL, and each dilution was tested in 25 replicates.^12^ The LOD was defined as the lowest HCV RNA level detected 95% of the times.^13^

### 2.6 Interference and cross‐reactivity study

The interference experiment was carried out using the HCV RNA interfering substance kit (Daan), which took HCV RNA‐positive serum (WHO international standard, NIBSC code: 06/102) and five kinds of interfering substances as raw materials to generate serums with 30 mg/dL bilirubin, 3.2 g/dL triglyceride, 30 g/dL hemoglobin, 6 g/dL albumin, or 18 g/L total immunoglobulin G, containing 2.21 × 10^5^ IU/mL HCV RNA or 2.77 × 10^3^ IU/mL HCV RNA. The two kinds of HCV RNA concentration serum with different interfering substances went through the HCV RNA quantitative assay to evaluate the effect of different interfering substances on the high concentration or low concentration HCV RNA‐positive serum.^14^ Moreover, the HCV RNA‐negative serums with 30 mg/dL bilirubin, 3.2 g/dL triglyceride, 30 g/dL hemoglobin, 6 g/dL albumin, or 18 g/L total immunoglobulin G were also tested. Five specimens positive for hepatitis B virus (HBV), five specimens positive for cytomegalovirus (CMV), and five specimens positive for Epstein‐Barr virus (EBV) and dengue virus (DV) were tested for the cross‐reactivity study. The specimens used for cross‐reactivity study were negative for HCV RNA.^15^

### 2.7 Comparison between Daan HCV RNA quantitative assay and COBAS AmpliPrep/COBAS TaqMan HCV Quantitative Test, v2.0

One hundred and ninety eight specimens quantitated by COBAS AmpliPrep/COBAS TaqMan HCV Quantitative Test, v2.0 in the First Affiliated Hospital, Sun Yat‐sen University from April to October 2019 were collected to evaluate the clinical performance of Daan HCV RNA quantitative assay. The serum was stored at −80℃ until detected by Daan HCV RNA quantitative assay.

### 2.8 Statistical analysis

HCV RNA quantities were log10 transformed before analysis. The variability among Daan HCV RNA quantitative assays was presented by the standard deviation (SD) and the percent coefficient of variation (CV) for the log10 transformed values. Linearity was analyzed by linear regression using the data from 3 replicates of serial dilutions. Deming regression analysis, Bland‐Altman analysis and Spearman's correlation coefficient were used to compare agreement of quantitative results for the positive specimens obtained by the two methods. Microsoft Excel, GraphPad Prism 6.0 (GraphPad Software), and MedCalc (MedCalc Software) were used to perform statistical analyses.

## 3 RESULTS

### 3.1 Precision and Accuracy

The S_within_, B, and S_total_ of the three concentrations of HCV RNA were calculated and shown in Table 1. The manufacturer's claim with‐run coefficient of variation (CV_within_) and total coefficient of variation (CV_total_) were both 5%. Thus, the σ_within_ and σ_total_ for 6.11 log IU/mL, 4.22 log IU/mL, and 2.32 log IU/mL HCV RNA were 0.31, 0.21, and 0.12, respectively. Compared with the σ_within_ and σ_total_, S_within_ and S_total_ of 6.11 log IU/mL and 4.22 log IU/mL were much less than the σ_within_ and σ_total_, indicating excellent precision of high and medium concentrations of HCV RNA. However, the S_within_ and S_total_ of 2.32 log IU/mL were close to the σ_within_ and σ_total_, suggesting the precision of low concentration HCV RNA was inferior to that of high and medium concentrations and variation was larger among assays with low concentration of HCV RNA. The SDs and CVs for different concentrations of HCV RNA were shown in Table 2. The CVs of 10^5^, 10^4^, 10^3^, and 10^2^ IU/mL were all below 5%, while the CV of 50 IU/mL was 11%. The CVs of each concentration indicated excellent accuracy. Moreover, the variation intended to become larger with the concentration decreasing.

**Table 1:** Precision of the DAAN HCV RNA quantitative assay

Expected, IU/mL	Log_10_ IU/mL	Daily Mean ± SD (log IU/mL)	Grand Mean ± SD (log IU/mL)	S_within_	B	S_total_
1.30 × 10^6^	6.11	6.36 ± 0.07	6.28 ± 0.15	0.13	0.01	0.15
		6.21 ± 0.14				
		6.42 ± 0.04				
		6.21 ± 0.15				
		6.21 ± 0.19				
1.66 × 10^4^	4.22	4.02 ± 0.07	4.09 ± 0.08	0.07	0.004	0.09
		4.09 ± 0.06				
		4.03 ± 0.07				
		4.15 ± 0.05				
		4.16 ± 0.04				
2.10 × 10^2^	2.32	2.53 ± 0.09	2.57 ± 0.11	0.11	0.001	0.10
		2.57 ± 0.09				
		2.54 ± 0.10				
		2.59 ± 0.12				
		2.63 ± 0.12				

Abbreviations: B, variance term; SD, standard deviation; S_total_, total precision; S_within_, within‐run precision.

**Table 2:** Accuracy of the DAAN HCV RNA quantitative assay

Expected, IU/mL	Log IU/mL	Mean	SD	CV (%)
10^5^	5	4.90	0.06	1.26
10^4^	4	3.84	0.04	1.08
10^3^	3	3.07	0.10	3.10
10^2^	2	2.16	0.09	3.95
50	1.7	1.62	0.18	11

Abbreviations: CV, coefficient of variation; SD, standard deviation.

### 3.2 Linearity and limit of detection (LOD)

**Figure 1:**
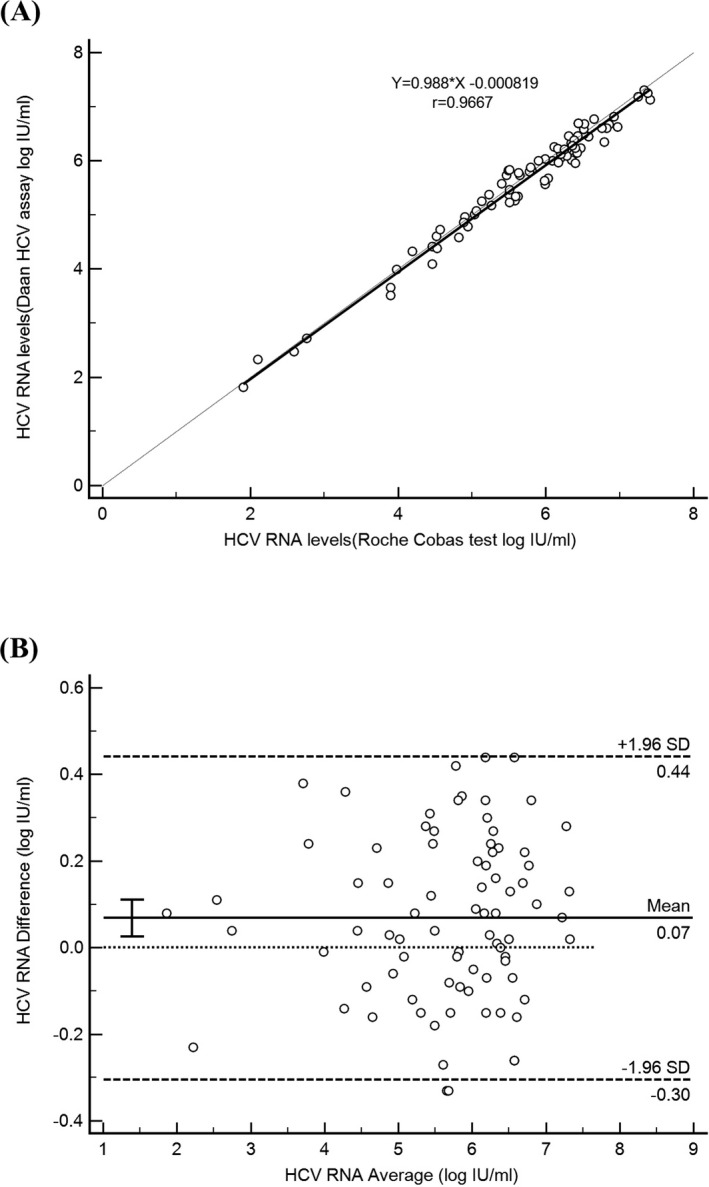
Linearity for Daan HCV RNA quantitative assay. Data are plotted as mean + standard error for each assay

As shown in Figure 1, the Daan HCV RNA quantitative assay exhibited a linear response from 1.3 log IU/mL to 8 log IU/mL. The equation for the linear regression line was y = 1.034 × x −0.1351, and the slope of 1.034 had a 95% confidence interval of 0.9889 to 1.080, including 1.00. The *R* ^2^ values for the linear goodness of fit were 0.9976. The HCV RNA detection rates for nominal HCV RNA concentrations of 50, 20, 15, and 10 IU/mL were 100%, 100%, 100%, and 84%, respectively. The specific HCV RNA quantitative results were shown in Table S1. The LOD was defined as the lowest HCV RNA level detected 95% of the times. As a result, the LOD was 15 IU/mL.

### 3.3 Interference and cross‐reactivity study

The results of the Daan HCV RNA quantitative assay for HCV RNA‐positive serum with different interfering substances were shown in Table 3. All the results were in the expected range, indicating bilirubin(up to 30 mg/dL), triglyceride(up to 3.2 g/dL), hemoglobin (up to 30 g/dL), albumin (up to 6 g/dL), or total immunoglobulin G (up to 18 g/L) did not influence the accurate detection of high concentration or low concentration HCV RNA‐positive serum. The negative serums with different interfering substances above were all tested negative. The Daan HCV RNA quantitative assay had not detected HCV RNA in the specimens positive for HBV, CMV, EBV, or DV. Thus, Daan HCV RNA quantitative assay showed no cross‐reactivity with HBV, CMV, EBV, and DV infections.

**Table 3:** HVC RNA quantitative results with different interferents

Expected (IU/mL)	Log IU/mL	Expected range (Mean ± 2SD log IU/mL)	Interferent	Interferent concentration	Mean ± SD (log IU/mL)
2.21 × 10^3^	3.34	2.83‐3.87	/	/	3.24 ± 0.09
			Bilirubin	30 mg/dL	3.06 ± 0.10
			Triglyceride	3.2 g/dL	3.16 ± 0.05
			Hemoglobin	30 g/dL	3.02 ± 0.02
			Albumin	6 g/dL	3.21 ± 0.08
			Total immunoglobulin G	18 g/L	3.19 ± 0.15
2.77 × 10^5^	5.44	4.94‐5.95	/	/	5.59 ± 0.02
			Bilirubin	30 mg/dL	5.56 ± 0.06
			Triglyceride	3.2 g/dL	5.62 ± 0.02
			Hemoglobin	30 g/dL	5.49 ± 0.01
			Albumin	6 g/dL	5.53 ± 0.11
			Total immunoglobulin G	18 g/L	5.59 ± 0.01

Abbreviation: SD, standard deviation.

### 3.4 Agreement between Daan HCV RNA quantitative assay and COBAS AmpliPrep/COBAS TaqMan HCV Quantitative Test

**Figure 2:**
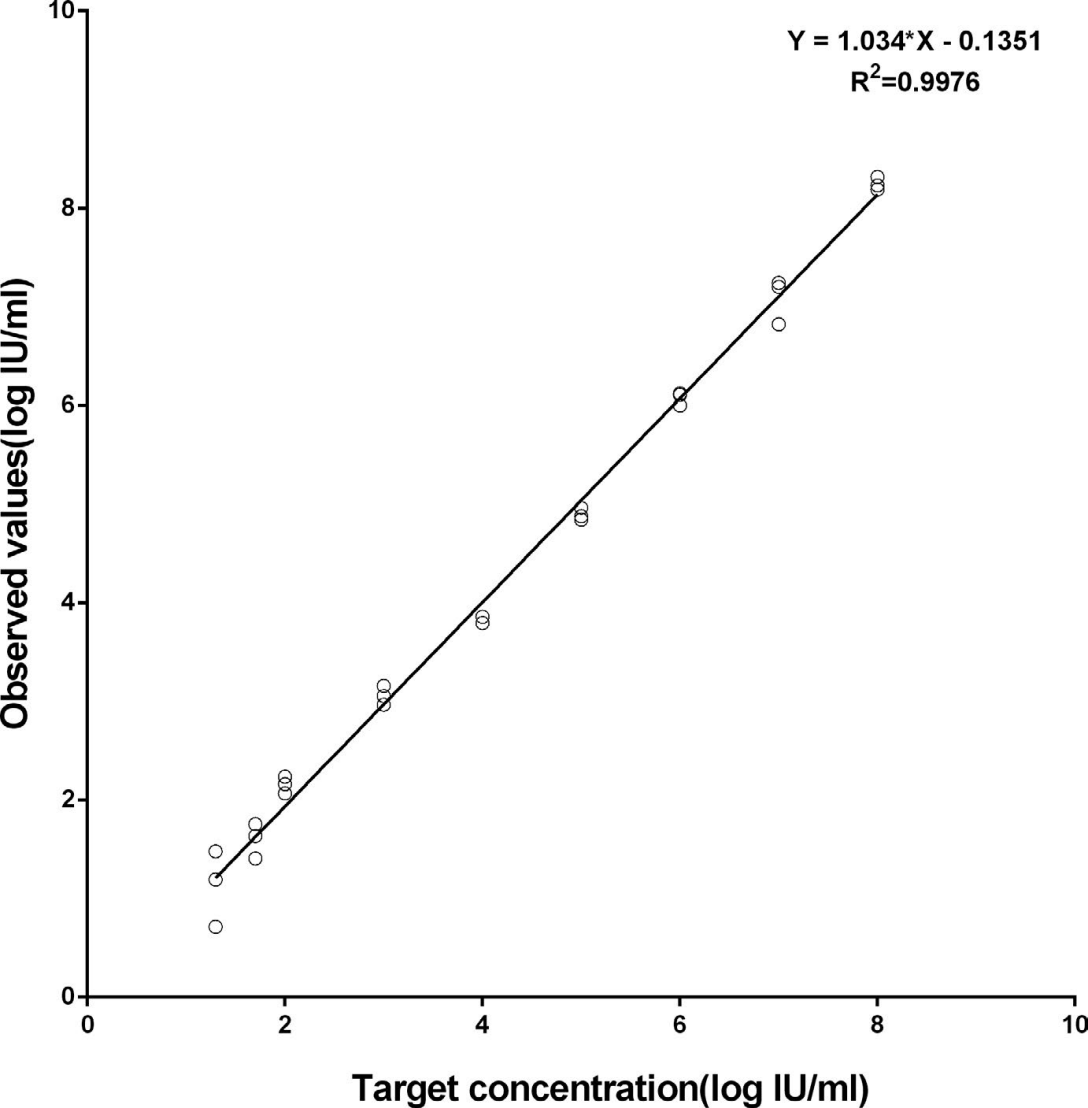
Deming linear regression analysis of HCV RNA levels and Bland‐Altman plot of data for all the clinical specimens showing the bias between the Roche Cobas test and Daan HCV assay. A, Deming linear regression analysis of HCV RNA levels for 81 serum specimens. The fitted regressions are represented with solid line. B, Agreement between the HCV RNA quantification by plotting the differences between the Roche Cobas test and Daan HCV assay averages of the two techniques using the Bland‐Altman analysis. Continuous line with the 95% limits of the agreement represents the mean bias between the two tests, and dashed lines represent the 95% confidence interval

Among 81 specimens tested positive by COBAS AmpliPrep/COBAS TaqMan HCV Quantitative Test, all were tested positive by the Daan HCV RNA quantitative assay. Of the 117 specimens tested negative by the Roche Cobas test, 115 specimens were tested negative by the Daan HCV RNA quantitative assay. The HCV viral load of the inconsistent samples was both below the LOD of Roche Cobas test and the Daan HCV RNA quantitative assay (both 15 IU/mL). Comparable performance of quantitative detection was illustrated by Deming regression analysis and Bland‐Altman analysis. A good correlation was observed between the two assays (Figure 2A). The median (IQR) HCV RNA concentration for Daan HCV RNA quantitative assay and the Roche Cobas test were 5.63 (1.82‐7.31) log IU/mL and 5.7 (1.9‐7.41) log IU/mL, respectively. Good agreement was observed by Bland‐Altman analysis between the two assays (Figure 2B), showing 77 quantitative results were within 95% limit of agreement among the 81 HCV RNA‐positive specimens. The mean difference (bias ± SD) between the Daan HCV RNA quantitative assay and the Roche Cobas test was −0.07 ± 0.37 log IU/mL.

## 4 DISCUSSION

It is necessary for an HCV RNA quantitative assay to provide reliable results and wide range of quantification because the chronically infected patients had wide ranges of HCV viral load.^16^ Moreover, broad‐range quantification was needed to monitor the response of the HCV‐infected patients to undetectable therapy levels of virus load.^17^ The S_within_ and S_total_ of the Daan HCV RNA quantitative assay for each verified concentration had reached the standard claimed by the manufacturer, demonstrating excellent precision of the quantitative assay. The greatest variability was observed with the low (2.32 log IU/mL) HCV RNA concentration which may be caused by RNA degradation. According to the results of linearity, the Daan HCV RNA quantitative assay has a measurable range of at least eight orders of magnitude, generating adequate linearity up to 8 log IU/mL (*R* ^2^ = 0.9976). The LOD of Daan HCV RNA quantitative assay was 15 log IU/mL which was comparable with COBAS AmpliPrep/COBAS TaqMan HCV Quantitative Test whose LOD was also 15 log IU/mL.^18^ Meanwhile, the HCV RNA quantitative assay with a 15 IU/mL limit of detection has been advocated by the Centers for Disease Control of United States and European Society of Liver Diseases as the confirmation test for HCV infection.^19^ What's more, 700 μL serum or plasma was needed for COBAS AmpliPrep/COBAS TaqMan HCV Quantitative Test while only 200 μL serum or plasma was needed for the Daan HCV RNA quantitative assay, suggesting the Daan HCV RNA quantitative assay needed less specimen volume to get a comparable performance. What's more, the Daan HCV RNA quantitative assay was of high specificity for it did not display any interference with commonly encountered conditions and other viral illnesses. From all above, the DAAN quantitative showed good precision and accuracy and exhibited a wide range of quantification, a comparable LOD and high specificity, indicating its excellent analytical performance. Moreover, our study was the first to evaluate this recently developed and certified diagnostic kit for quantification of hepatitis C virus RNA.

Good agreement was observed between the Daan HCV RNA quantitative assay and COBAS AmpliPrep/COBAS TaqMan HCV Quantitative Test on clinical specimens, indicating good clinical performance of the DAAN HCV RNA quantitative assay. Positive and negative agreement between them was 99%, suggesting a re‐baseline of patients is not needed when switching to the Daan HCV RNA quantitative assay for HCV testing. The Daan HCV RNA quantitative assay is a good candidate for diagnosing HCV and monitoring HCV viral load of patients during HCV therapy. From the comparison results, we could find that the specimen sets were enriched to 4‐7 log IU/mL viral load ones. The samples maybe tended to be uniform because of the homogeneous patient population. During the HCV RNA extraction, it could be carried out on automatic nucleic acid extractor platform Smart 32, which could avoid labor‐intensive manual extraction and reduce artificial error. Moreover, the price for per Daan HCV RNA quantitative assay was almost a quarter of that for the Roche Cobas test, making it affordable in many central laboratories in developing countries. With the advantage of low price and satisfactory analytical performance and clinical performance, the Daan HCV RNA quantitative assay would be a good alternative choice for the diagnosis and monitoring HCV RNA. However, the performance evaluation of the assay was carried out in only one central laboratory in China. Further studies on the performance evaluation of the Daan HCV RNA quantitative assay should be conducted at regional and peripheral level laboratories.

In summary, evaluation of the performances of the Daan HCV RNA quantitative assay confirmed the excellent characteristics of the assay regarding the precision, accuracy, linearity, anti‐interference ability, and good agreement with the Roche assay, making it a good alternative choice for the diagnosis and monitoring of HCV infection in developing countries.

## Supporting information

TableS1
