# Supplementary material for: Performance evaluation of the DAAN HCV assay for quantification of hepatitis C virus RNA and its comparison with COBAS AmpliPrep/COBAS TaqMan HCV Quantitative Test, v2.0
Source: J Clin Lab Anal. 2020 Mar 13;34(7):e23280. doi: 10.1002/jcla.23280 (PMC7370725; doi:10.1002/jcla.23280)
Supplement: Supplementary file 1 — TableS1 [file JCLA-34-e23280-s001.docx]

Calculate the with-run precision from the following formula^1^:

Calculate the variance term B for the daily means from the formula^1^:

Calculate the S_total_ precision from the following formula^1^:

$$s_{total}=\sqrt{\frac{n-1}{n}\times s_{within}^{2}+B}$$

Where:

**∑** indicates that the terms to the right of **∑** are to be summed

D = total number of days

n = total number of replicates per day

*x*_di_= result for replicates per day

$\bar{x}_{d}$= average of all results for day d

n = number of replicates per run

$x$= average of all results

σ_within_ =$CV_{within}\bullet x$

σ_total_ =$CV_{total}\bullet x$

where CV_within_ and CV_total_ is the manufactuer’s claim with-run and total coefficient of variation.

**Reference:**

1. CLSI. User Verification of performance for precision and Trueness；approved guideline-second edition. In. *CLSI documents EP 15-A2*: Wayne, PA, USA: CLSI; 2008.

**Supplementary Table 1 HCV RNA quantitative results of 50, 20, 15, and 10 IU/ml dilutions**

| Target  (IU/ml) | Detected  value (IU/ml) | Target  (IU/ml) | Detected  value (IU/ml) | Target  (IU/ml) | Detected  value (IU/ml) | Target  (IU/ml) | Detected  value (IU/ml) |
| --- | --- | --- | --- | --- | --- | --- | --- |
|  | 67.16 |  | 36.67 |  | 13.43 |  | 34.94 |
|  | 76.94 |  | 14.14 |  | 27.10 |  | 22.93 |
|  | 69.86 |  | 18.35 |  | 11.87 |  | 10.20 |
|  | 65.55 |  | 28.99 |  | 19.20 |  | 14.33 |
|  | 71.26 |  | 23.01 |  | 16.38 |  | 5.80 |
|  | 44.92 |  | 36.31 |  | 24.41 |  | 16.60 |
|  | 31.04 |  | 29.16 |  | 10.91 |  | 12.57 |
|  | 79.56 |  | 39.01 |  | 25.50 |  | 15.39 |
|  | 66.58 |  | 21.69 |  | 25.43 |  | 2.25 |
|  | 44.61 |  | 44.71 |  | 21.36 |  | 8.77 |
| 50 | 33.88 | 20 | 22.45 | 15 | 5.09 | 10 | 7.06 |
|  | 70.17 |  | 22.42 |  | 17.09 |  | 8.91 |
|  | 92.45 |  | 7.66 |  | 14.10 |  | 19.64 |
|  | 41.28 |  | 18.95 |  | 19.63 |  | 14.24 |
|  | 51.00 |  | 19.87 |  | 11.94 |  | 8.74 |
|  | 83.42 |  | 23.04 |  | 22.58 |  | 13.29 |
|  | 49.10 |  | 20.64 |  | 20.32 |  | 14.59 |
|  | 61.25 |  | 14.16 |  | 32.69 |  | 4.71 |
|  | 44.31 |  | 9.34 |  | 21.49 |  | 9.54 |
|  | 74.25 |  | 18.01 |  | 18.30 |  | 25.11 |
|  | 71.05 |  | 13.35 |  | 13.10 |  | 29.99 |
|  | 64.52 |  | 10.94 |  | 28.79 |  | Not detected |
|  | 50.34 |  | 40.69 |  | 23.00 |  | Not detected |
|  | 47.70 |  | 11.66 |  | 24.36 |  | Not detected |
|  | 58.37 |  | 20.75 |  | 25.57 |  | Not detected |
| Detection  rate (%) | 100 |  | 100 |  | 100 |  | 84 |
